# Efficacy of biorational insecticides against *Bemisia tabaci* (Genn.) and their selectivity for its parasitoid *Encarsia formosa* Gahan on *Bt* cotton

**DOI:** 10.1038/s41598-021-81585-x

**Published:** 2021-01-22

**Authors:** Muhammad Dildar Gogi, Ali Hassan Syed, Bilal Atta, Muhammad Sufyan, Muhammad Jalal Arif, Muhammad Arshad, Ahmad Nawaz, Muhammad Ahsan Khan, Adeel Mukhtar, Oscar Emanuel Liburd

**Affiliations:** 1 Department of Entomology, University of Agriculture, Faisalabad, Punjab Pakistan; 2 Rice Research Institute, Kala Shah Kaku, Punjab Pakistan; 3 Institute of Pest Warning and Quality Control of Pesticides, Sahiwal, Punjab Pakistan; 4 Entomology and Nematology Department, University of Florida, Gainesville, FL USA

**Keywords:** Entomology, Environmental sciences

## Abstract

The toxicity of seven biorational insecticides [five insect growth regulators (Buprofezin, Fenoxycarb, Pyriproxyfen, Methoxyfenozide, and Tebufenozide) and two oil-extracts of neem and bitter gourd seeds] against *Bemisia tabaci* and their selectivity for its parasitoid, *Encarsia formosa* were evaluated in laboratory and field conditions for 2 years (2018–2019) in Pakistan. Toxicity results demonstrate that Pyriproxyfen, Buprofezin, and Fenoxycarb proved to be effective (80–91% mortality and 66.3–84.2% population-reduction) against *B. tabaci* followed by Methoxyfenozide, Tebufenozide (50–75% mortality and 47.8–52.4% population-reduction), and then oil-extracts of neem and bitter gourd (25–50% mortality and 36.5–39.8% population-reduction) in the laboratory [72 h post-application exposure interval (PAEI)] and field trails (168 h PAEI), respectively. All tested biorationals, except Methoxyfenozide [(slightly-harmful/Class-II), i.e., causing mortality of parasitoids between a range of 25–50%] and Tebufenozide [(moderately-harmful/Class-III), i.e., causing mortality of parasitoids between the ranges of 51–75%], proved harmless/Class-I biorationals at PAEI of 7-days in the field (parasitism-reduction < 25%) and 3-days in the lab (effect < 30%). In laboratory bioassays, exposure of parasitized-pseudopupae and adult-parasitoids to neem and bitter gourd oils demonstrated that these compounds proved harmless/Class-I biorationals (< 30% mortality). Alternatively, Pyriproxyfen, Buprofezin, Fenoxycarb, Methoxyfenozide, and Tebufenozide were slightly-harmful biorationals (30–79% mortality) against the respective stages of *E. formosa*. We conclude that most of the tested biorationals proved harmless or slightly harmful to *E. formosa*, except tebufenozide after PAEI of 7-days (168 h) in the field and, therefore, may be used strategically in Integrated Pest Management (IPM) of *B. tabaci*.

## Introduction

Whitefly, *Bemisia tabaci,* is a serious pest of cotton and many other vegetables and orchard crops^1–4^. It starts feeding on cotton plants from the early stage of the crop up to maturity, causing a significant decline in boll production, which can reach up to 50% in the case of severe attacks^1–3^. It also impairs photosynthesis by depositing honeydew on leaves, which become blackish due to sooty mold growth^3,5^. The leaf curl virus transmitted by this species also causes a 20–40% reduction in cotton bales^2,3,6,7^. In Pakistan, *B*. *tabaci* is parasitized by two parasitoids, *Encarsia formosa* (Hymenoptera: Aphelinidae) Gahan and *Eretmocerus* species (Hymenoptera: Aphelinidae) in the cotton crop^1–4^. Up to 10–14% parasitism of *B*. *tabaci* has been reported in cotton in Pakistan^1–3^. Sucking insect pests, including whiteflies in *Bacillus thuringiensis* (*Bt*) cotton are managed by foliar applications of synthetic insecticides in Pakistan^3,8^. A variety of insecticides have been tested against whiteflies in the past. These insecticides include carbamates (carbosulfan and aldicarb), pyrethroids (fenpropathrin, cypermethrin, and lambda-cyhalothrin), neonicotinoids (diafenthiuron, acetamiprid, imidacloprid, thiacloprid, nitenpyram, and thiamethoxam), insecticide-mixtures [NOVASTAR (bifenthrin + abamectin) and DELTAPHOS (deltamethrin + triazophos)], sulfoximines (sulfoxaflor), insect growth regulators [(IGRs) (buprofezin, pyriproxyfen)] and Tetronic/Tetramic acid derivatives (Spirotetramat, spiromesifen)^4,9–20^. However, indiscriminate use of insecticides has resulted in the rapid development of resistance to sucking insects, including whitefly populations and adverse effects on their natural enemies^3,8,14,17–20^. In plants, conventional and systemic IGRs insecticides are biotransformed by nonmicrosomal (IGRs) or microsomal biotransformation reactions (hydrolysis) and associated enzymes, which greatly vary among types, species, and varieties of plants (crops)^21,22^; hence, their impacts on non-target arthropods greatly vary on a variety of plant species^4,21^. *Bt* crops have great potential to augment the role of biocontrol in integrated pest management systems. Conventional and *Bt* cotton cultivars have different production technology [different sowing-time, fertilizer (nutrient) and water requirements, etc.] and influence various biotic stresses and exogenic molecules differently due to differences in their genetic background and physiology/metabolism^22^. Variation in the agronomic aspects of their production technologies (nutrients, irrigation, etc.) affects the quantity and quality of specialized-metabolite biosynthesis differently in *Bt* and non-*Bt* crops^23,24^. Some reports also advocate that *Bt* crops’ physiology and metabolism affect the toxicity of systemic insecticides against pest and its selectivity against natural enemies through metabolic interaction of *Bt*-toxin (proteins) or its metabolites (protoxins) with insecticide molecules within the plant tissues^25^. A few in-vivo studies have indicated that a combination of synthetic and microbial insecticides (including entomopathogenic fungi and bacteria) can have additive, antagonistic or synergistic toxicity effects against target insects^26–28^. However, research on the aforementioned lines has not been reported on *Bt* cotton. The use of synthetic biorationals, including IGRs and botanical extracts for controlling whiteflies has increased in Pakistan, but their efficacy against *B. tabaci* and selectivity for *E. formosa* in *Bt* cotton has yet to be explored.

IGRs are highly selective insecticides having biorational impacts on pests^8,29–32^. As biorational insecticides, they disrupt hormonal regulation and ultimately result in abnormal molting, growth, and development in several insects. They are used for the control of many insect species acting as ecdysone agonists (tebufenozide and halofenozide), juvenile hormone mimic (pyriproxyfen) and chitin synthesis inhibitors (benzoylphenylureas and buprofezin). Buprofezin (2-*ter*-butylimino-3-isopropyl-5-phenylperhydro-1, 3, 5-thiadiazin-4-one) possesses strong contact and stomach toxicity and has been reported as less toxic and highly selective for natural enemies (predators and parasitoids), fish, birds, and silkworms. Buprofezin inhibits chitin synthesis, interrupts metabolism, and causes mortality without molting or due to deformed molting^4,33^. It has been evaluated against whiteflies on cotton^4,34^, planthoppers on rice^35^, jassids on brinjal^36^, and mealybugs on grapes^37,38^ and cotton^39^. Fenoxycarb acts as a juvenile hormone mimic and has been documented as an effective molecule for controlling various insect pests infesting economic crops, ornamentals, and fruits. It interrupts the metamorphosis, growth, and development of all insect life stages and inhibits oogenesis and the development of ovaries^40^. Pyriproxyfen also possesses juvenile hormone mimic properties but has no indirect lethal effects on female fertility, egg viability, sex ratios of different predators, and parasitism capacity of various parasitoids^41,42^. Tebufenozide and methoxyfenozide as IGRs act both as stomach and contact poisons with a 3-week residual activity period. Both result in mortality by a failure of feeding in target insects or by disrupting the normal process of molting, specifically in lepidopteran pests^43,44^. These IGRs have been documented safe for honeybees and most of the other natural enemies^45^.

IGRs have been reported as highly compatible insecticides with certain species of parasitoids of *B.* *tabaci*; hence, biocontrol efficacy of *E. formosa* can be complemented with strategic application of IGRs in greenhouse and field IPM program of crops^4,46^. However, before devising IGRs and parasitoid-based IPM strategy for *B.* *tabaci*, it is essential to investigate the non-target effects of IGRs on various biological parameters of *E. formosa*^46^*.* Some studies have shown that different development stages of Hymenopterous parasitoids respond differently when exposed to IGR treatments^41,42,46,47^. For example, the results of a leaf and glass-vial residue bioassays confirmed that pyriproxyfen, fenoxycarb, and buprofezin caused no considerable effects on the survival of adults of *E. formosa*. The residues of pyriproxyfen and fenoxycarb resulted in a slight reduction in parasitism compared with buprofezin. However, pyriproxyfen, fenoxycarb, and buprofez caused a significant decrease in the rates of parasitoids’ adult emergence^46^. *Encarsia formosa* females emerging from the buprofezin-treated pupae exhibited shorter lifespan (0.7–1.7 days), lower parasitism rate (11.7%), and less adult emergence (11.6–17.8%) than control treatment^47^. The results of lab bioassay of pyriproxyfen against three species of endoparasitoids [*Enearsia pergancliella, E. transvena* (Timberlake) and *E. formosa* Gahan] and their host (*Bemisia argentifolii* Bellows & Perring) show that pyriproxyfen was effective against *B. argentifolii,* harmless to *E. pergandiella,* moderately harmless to *E. transvella,* and moderately harmful (toxic) to *E. formusa,* especially pupae^48^. Based on the mortality scale specified by the International Organization for Biological Control (IOBC), pyriproxyfen proved harmful, whereas buprofezin and fenoxycarb proved slightly-moderately harmful to larvae and harmless to pupae of *E. formosa* when assessed through leaf and glass-vial residues bioassays^46^.

Similarly, botanicals have been reported as environmentally safe compounds with lower toxic effects on mammals, natural enemies and pollinators. These compounds are considered important components of IPM programs of any crop. Extracts of neem (*Azadirachta indica* A.) and bitter gourd (*Momordica chrantia* L.) were effective for the management of sucking insect pests of cotton^49^. The most prominent constituent of the seed-kernels, leaves, and bark of neem is azadirachtin, which has a strong insecticidal property. It not only induces some behavioral effects like antifeedency, repellency, etc. in insects by interfering with chemoreceptory mechanisms, but also disrupts the growth mechanism of insects through interfering with the neuroendocrine control of molting and edysis^50^. *Momordica charantia*, is a tropical to sub-tropical vine in the family Cucurbitaceae. The plant produces triterpenoid compounds in the leaves, fruits and seeds, which have been reported to have antifeedant and oviposition-inhibition properties on arthropods^51–54^. However, the impact of these biorational insecticides is not well understood on the whitefly and its parasitoid in *Bt* cotton.

A comprehensive understanding of the influence of environmental factors (temperature, relative humidity, photoperiod, etc.) on the interaction between pest and its bioagent is of utmost importance for successful and sustainable implementation of any biological control program^2,3,5,55,56^. Female *E. formosa* exhibits the maximum parasitism on greenhouse whitefly *Trialeurodes vaporariorum* (Westwood) at 25 °C and no parasitization below 12 °C or above 40 °C^55^. Treatment involving simulated summer environment [82–83.6 W/m^2^ (high light intensity), L 16: D 8 h (long photoperiod), 24 °C (temperature)] administrated significantly higher parasitism of *T. vaporariorum* by *E. formosa* and *E. eremicus* as compared to treatment involving simulated winter environment [10.8–11.1 W/m^2^ (low light intensity), L 8: D 16 h (photoperiod), 20 °C (temperature)]^56^. Biological behavior and response of biocontrol agents toward their host/prey under insecticides’ stress is also influenced by several environmental factors (humidity, temperature, rainfall, wind-speed, etc.) which are responsible for the post-application degradation and dissipation of insecticides^4,57^. The results reviewed in some research reports show that a steady increase in temperature (beyond 30 °C) and decrease in relative humidity (below 70%) cause gradual degradation of IGRs’ residues on plants or in other media, reduction in their half-life and resultantly reduction in their toxicity against parasitoids/predators and its host/prey^4,57,58^.

Based on the aforementioned facts, it is concluded that *B. tabaci* is a major cotton pest parasitized by *E. formosa* and *Eretmocerus* species and foliar application of insecticides is the most important pest management technique for *B. tabaci* and/or sucking pest complex in Pakistan. The conventional insecticides used for the management of *B. tabaci* and/or sucking pest complex are not target specific and suppress the population of their respective predators and parasitoids in the agro-ecosystem. Synthetic-biorationals, including IGRs and botanical-extracts are currently being researched for the management of *B. tabaci* and/or sucking pest complex. However, most of the published work highlights the research studies conducted on various insecticides in other countries on fruit-crops and crops other than *Bt*-cotton, where there are differences in environmental conditions and technology. It has been reported that a biorational tool is effective on some crops, but not on others because of the variation in several factors [like plant-species (crop types), plant development-stages, physiochemical properties of chemicals, formulation of chemicals, application methods and climatic conditions (humidity, temperature, rainfall, wind-speed, etc.)] which are responsible for the degradation and dissipation of biorational insecticides after their spray on plants^4,57^. For example, foliar application of ASSAIL (acetamiprid) 20% SL) @ 300 mL/ha demonstrated a significant variation in *B. tabaci* population reduction on five cotton varieties including super-98 (65% reduction), BH-163 (98% reduction), VH-159 (73% reduction), NIAB-884 (83% reduction) and NIAB-101 (68% reduction) at 72 h post-application interval^9^.

Whiteflies currently threaten cotton production in Pakistan; therefore, it is important to identify biorational tools that are readily available for the management of whitefly populations. Again, management programs that are developed for other countries in other crops may not relate to the subtropical and relatively arid environmental conditions in Pakistan where cotton is such an important crop to the country and the citizens of Pakistan. There is a need for an extensive investigation on the efficacy of such biorationals against whitefly and their selectivity against natural enemies. The current study evaluated seven biorational insecticides including two oil-extracts (Neem-extract, bitter gourd-extract) and five IGRs (buprofezin, fenoxycarb, pyriproxyfen, methoxyfenozide, and tebufenozide) for their efficacy against *B. tabaci* and selectivity for its parasitoid *E. formosa* on *Bt* cotton in laboratory and field conditions. The findings would have applications in developing countries where the goal is to use fewer toxic pesticides for protecting humans, natural enemies, pollinators, and the environment.

## Materials and methods

### Collection and preparation of botanical extracts

For the preparation of botanical extracts of neem (*A. indica* L.) and bitter gourd (*M. charantia* L.), one kilogram each of fresh neem and bitter gourd seeds was purchased from a local market and rinsed thoroughly in distilled water. The seeds were then dehulled, put in paper bags, and subsequently dried inside an oven at 50 °C until a constant weight was obtained. The dried seeds of both neem and bitter gourd were cooled down to room temperature and ground into a powder in a grinding mill separately. After grinding, the powder of each was stored in separate air-tight containers and labeled. For extraction of oil, 50 g of neem seed powder was processed through soxhelt extraction using n-Hexane as the solvent. The miscella of n-hexane and neem oil obtained by soxhelt extraction was purified at 68 °C (boiling point of n-hexane) by evaporation of n-hexane inside the rotary evaporator. Neem oil dissolves easily in water and does not need a surfactant. The same procedure was followed for the extraction of oil from bitter gourd seeds. For laboratory and field experiments, 1000 ppm of each extract was prepared by dissolving 1.0 mL of extract in 1L of water.

### Insect growth regulators

Five IGRs, buprofezin (chitin synthesis inhibitor), fenoxycarb, pyriproxyfen (juvenile hormone mimics), methoxyfenozide, and tebufenozide (molting hormone agonists) were purchased from a local market and evaluated at their field recommended doses by using a 3.5-gallon knapsack sprayer with a Teejet hollow cone spray cores D3 disk (Spraying systems Co., Keystone Heights, FL USA) spraying 1380-L of water/ha for each application in the field and by an atomizer in laboratory studies. The field recommended dose rate for buprofezin (Agrochemicals, Karachi, Pakistan), fenoxycarb (Syngenta, Karachi, Pakistan), pyriproxyfen methoxyfenozide (Agrochemicals, Karachi, Pakistan) and tebufenozide (Valent Biosciences, Lahore, Pakistan) were 500, 200, 120, 200 and 160 mL/0.4 ha, respectively.

### Laboratory evaluation

The leaves infested with pseudopupae of whitefly were clipped from untreated cotton plants potted in earthen-pots kept in the field for natural whitefly infestation and its parasitism by *E. formosa*. The leaves were placed on moistened filter paper in Petri dishes and maintained at 25 ± 2 °C and 75 ± 5% relative humidity in an incubator until the adult parasitoids emerged. The emerged parasitoids were aspirated and kept in glass jars provided with sponge rubber foam strips soaked with adult diet (honey, distilled water, and yeast-powder in 1:9:1 ratio) for two days. After two days, ten parasitoids (5 males and 5 females) were aspirated and released into parasitizing-chambers. Each parasitizing-chamber consisted of potted cotton (*Bt*-cotton *var.* Lalazar) infested with unparasitized pseudopupae, larval instars, and twenty whitefly adults. The experimental layout was a Completely Randomized Design (CRD) with three replications. The total number of pseudopupae and other larval instars present on the leaves inside the chamber were also counted. The cotton plant inside the chamber was sprayed with biorational insecticides using the atomizer. Neem was applied at the manufactured labeled rate (60 mL to 3. 8 L) without a surfactant. Parasitoids were released into the chamber 24 h after treatment. The numbers of dead whitefly adults, larval instars, and male/female adult parasitoids were counted after the three-day treatment and data obtained were transformed into percent corrected mortality over control using Henderson–Tilton’s formula.

Observations were made 2 weeks after treatment to count dark-colored parasitized whitefly pseudopupae to determine the rate of parasitism, and percent parasitism was calculated. The black-colored parasitized pseudopupae were also kept under observation for 1 week and the number of *E. formosa* adults that emerged from parasitized pseudopupae were counted and the data obtained were transformed into percent emergence of adult *E. formosa* from parasitized pseudopupae. The rate of parasitism was calculated as the percent emergence of adult *E. formosa* from the parasitized pseudopupae. The parasitoid mortality percentage inside whitefly pseudopupae was computed by subtracting the adult emergence percentage from 100 and then transformed into percent corrected parasitoid mortality over control using Henderson–Tilton’s formula.

The laboratory experiment was duplicated in 2018 and 2019. Since there was no significant difference in parasitization rate or success rate of parasitism between the two years, data from the two experiments were pooled in statistical analysis.

The mortality scale specified by the IOBC to describe the effects of pesticides on beneficial species for lab studies (< 30% = harmless/Class-I; 30–79% = slightly harmful/Class-II; 80–99% = moderately harmful/Class-III; > 99% = Harmful/Class-IV)^59^ was used to ascertain the degree of toxicity or safety of tested insecticides for *E. formosa.*

### Field evaluation

*Bt*-cotton var. Lalalzar was sown in Randomized Complete Block Design (RCBD) with three replications per treatment plot measuring 34 × 54 m^2^. Sowing was done with a plant to plant distance of 45 cm and row to row distance of 90 cm. The treatments were applied at their field recommended doses when the whiteflies reached their economic threshold level (ETL). The rest of the agronomic practices were carried out uniformly in all treatments. Whitefly population was recorded 24 h before and 24, 48, 72 and 168 h post-application exposure interval (PAEI). The data were collected according to Frank and Liburd’s method of pest scouting^60^. In total, 10 plants were selected randomly from every replication and the population (both adults and nymphs) of whiteflies per leaf was recorded visually with the help of hand-lens from the upper, middle, and lower leaf of the first, second and third plants respectively. The collected whitefly population data were transformed into percent population reduction in whitefly samples collected at 24, 48, 72-and-168 h PAEI as compared to the whitefly population recorded 24 h before treatments’ application. The survival rate percentage was calculated.

In the parasitization experiment, five plants were tagged in each replication of each treatment and the numbers of parasitized and unparasitized whitefly pseudopupae were counted 24 h PAEI. The parasitized pupae were marked with a permanent marker and percent parasitism 24 h before the application was calculated. Then the number of newly parasitized pseudopupae of whitefly were counted and marked with permanent marker at 24, 48, 72, and 168 h PAEI. The percent reduction in parasitism due to biorational insecticides was computed for each PAEI using Sun–Shepard’s formula^61^.

The mortality/parasitism-reduction scale specified by the IOBC to describe effects of pesticides on the survival/performance of beneficial species for semi-field and field studies (< 25% = harmless/Class-I; 25–50% = slightly harmful/Class-II; 51–75% = moderately harmful/Class-III; > 75% = harmful/Class-IV)^59^ was used to describe the tested biorational insecticides as harmful or safe.

Five leaves with parasitized whitefly pseudopupae were removed from each treated and control plants. The parasitized pseudopupae present on each leaf were counted. The clipped leaves were placed on water-soaked and tissue-paper wrapped sponge adjusted in Petri dishes which were kept covered with perforated lid tightened with a rubber band. The Petri dishes were then placed inside the growth chamber maintained at 30 ± 2 °C and 65 ± 5% RH until the adult parasitoid emerged. This method was repeated for each PAEI. The emerging parasitoid adults from parasitized whitefly pseudopupae for each biorational insecticide and the control for each PAEI were counted and the data obtained were transformed into percent mortality and adult emergence of the parasitoid.

The field experiment was duplicated in 2018 and 2019. Since there was no significant difference in dependent variables between the 2 years, data from the two duplicated experiments were pooled in statistical analysis.

### Statistical analysis

Data on parasitism, and population reduction of *B. tabaci* in field studies, and mortality of *B. tabaci* and parasitisim and mortality of *E. formosa* in laboratory studies were analyzed using the General Linear Model (GLM) through analysis of variance (ANOVA) technique^62^ in SAS 2009^62^. The means among treatments were compared using Tukey’s Honestly Significant Difference (HSD) test at a probability level of 5%.

### Ethics approval and consent to participate

The authors agree to all the concerned regulations.

### Consent for publication

The authors agree to publish this scientific paper at the Scientific Reports.

## Results

### Impact of biorationals under field conditions

Insecticide applications had significant effects on *B. tabaci* population (Table 1), parasitism of whiteflies by *E. formosa* (Table 2), and the emergence of adult parasitoids (*E. formosa*) at 24, 48, 72, and 168 h PAEI (Tables 3, 4).

**Table 1:** Population reduction percentage of whitefly at different post-treatment intervals of different insecticides on *Bt* cotton under field conditions.

Treatments	Whitefly population reduction percentage (Mean ± SE)			
	24 h	48 h	72 h	168 h
Pyriproxyfen	44.16 ± 0.73 a	61.23 ± 2.91 a	84.18 ± 2.33 a	73.21 ± 3.43 a
Buprofezin	40.55 ± 0.77 b	57.31 ± 2.54 ab	73.45 ± 2.67 b	69.34 ± 3.65 ab
Fenoxycarb	36.28 ± 0.54 c	53.31 ± 2.76 abc	66.25 ± 2.23 c	55.30 ± 3.23 abc
Methoxyfenozide	32.39 ± 0.43 d	49.36 ± 2.66 bcd	52.41 ± 2.34 d	41.40 ± 3.12 abc
Tebufenozide	27.90 ± 0.88 e	44.88 ± 2.45 cde	47.84 ± 2.14 e	36.92 ± 3.11 abc
Neem	22.65 ± 0.71 f	41.55 ± 2.78 de	39.75 ± 2.16 ef	26.61 ± 3.22 bc
Bittergourd	19.47 ± 0.49 g	36.48 ± 2.93 f	32.57 ± 2.11 f	21.49 ± 2.33 c
Control	3.59 ± 0.33 h	5.25 ± 2.02 f	8.35 ± 2.22 g	4.22 ± 1.01 d
P value	0.0003	0.0001	0.00004	0.00002
F value	634.39**	50.17**	10.03**	17.90**
*df*	7^a^/13^b^	7^a^/13^b^	7^a^/13^b^	7^a^/13^b^

Means with the same lowercase letters are not significantly different at α = 0.05 (Tukey’s test).

*df* degree of freedom.

^a^Degree of freedom of treatment.

^b^Error degree of freedom.

**Highly significant at probability level of 5%.

**Table 2:** Percent parasitism of whitefly by *E. formosa* at different post-treatment intervals of different insecticides on *Bt* cotton under field conditions.

Treatments	Percent parasitism of whitefly by *E. formosa* (Mean ± SE)				
	Before application	24 h PAEI	48 h PAEI	72 h PAEI	168 h PAEI
Pyriproxyfen	35	12.5 ± 2.73 d (71.3 MH)	17.3 ± 3.21 d (65.8 MH)	55.2 ± 2.14 c (2.42 HL)	68.2 ± 4.13 c (− 15.3 HL)
Buprofezin	37	11.4 ± 1.77 d (75.1 HF)	14.6 ± 2.94 f (72.7 MH)	57.4 ± 2.55 c (3.73 HL)	71.5 ± 4.65 b (− 14.9 HL)
Fenoxycarb	38	8.6 ± 0.94 e (80.8 HF)	9.7 ± 1.36 f. (81.4 HF)	46.6 ± 1.67 d (19.6 HL)	64.6 ± 2.93 d (− 6.6 HL)
Methoxyfenozide	39	4.6 ± 0.73 f (89.9 HF)	5.4 ± 0.96 g (89.9 HF)	22.4 ± 1.44 e (62.5 MH)	35.3 ± 2.15 e (43.3 SH)
Tebufenozide	40	2.4 ± 0.48 f (95.0 HF)	3.3 ± 0.55 g (94.1 HF)	15.5 ± 1.25 f (75.3 HF)	24.6 ± 2.01 f (63.3 MH)
Neem	37	18.7 ± 1.71 c (59.2 MH)	24.6 ± 4.18 c (53.9 MD)	57.8 ± 2.98 c (3.1 HL)	68.5 ± 3.82 c (− 10.9 HL)
Bittergourd	36	21.5 ± 3.49 b (54.3 MH)	29.2 ± 3.97 b (46.6 SH)	60.4 ± 3.81 b (1.3 HL)	73.6 ± 5.73 b (− 15.4 HL)
Control	35	42.4 ± 4.33 a	55.4 ± 5.42 a	74.3 ± 4.62 a	86.4 ± 6.06 a
P value		0.00002	0.0006	0.00001	0.0004
F value		605.5**	1064.6**	1495.5**	1614.7**
*df*		7^a^/13^b^	7^a^/13^b^	7^a^/13^b^	7^a^/13^b^

Means with the same lowercase letters are not significantly different at α = 0.05 (Tukey’s test). Positive values in parenthesis indicate the corrected percent reduction while negative values inside parenthesis indicate corrected percent increase in parasitism over control at different PAEI. HL, SH, MH and HF abbreviations inside the parenthesis indicate Harmless/Class-I, Slightly-harmful/Class-II, Moderately-harmful/Class-III and Harmful/Class-IV insecticides, respectively).

*df* Degree of freedom.

^a^Degree of freedom of treatment.

^b^Error degree of freedom.

**Highly significant at probability level of 5%.

**Table 3:** Percent emergence and mortality of adult *E. formosa* when parasitized pseudopupae were exposed to different insecticides at different post-application exposure intervals on field cultivated *Bt* cotton.

Treatments	Emergence of adult *E. formosa* from field collected parasitized pseudopupae (%) (Mean ± SE)			
	24 PAEI	48 PAEI	72 PAEI	168 PAEI
Pyriproxyfen	25.2 ± 4.3 d (71.8 MH)	26.4 ± 6.2 d (70.4 MH)	58.2 ± 6.8 c (39.1 SH)	69.5 ± 13.1 d (27.1 SH)
Buprofezin	22.5 ± 3.9 e (74.9 MH)	22.9 ± 7.1 e (74.3 MH)	54.0 ± 6.1 cd (43.5 SH)	67.1 ± 12.8 e (29.6 SH)
Fenoxycarb	18.1 ± 3.1 f (79.8 HF)	19.9 ± 9.1 f (77.7 HF)	48.3 ± 5.6 de (49.4 SH)	55.3 ± 11.7 f (42.0 SH)
Methoxyfenozide	11.7 ± 2.8 g (86.9 HF)	12.3 ± 4.8 g (86.2 HF)	43.6 ± 5.1 ef (54.4 MH)	53.4 ± 10.6 g (44.0 SH)
Tebufenozide	8.4 ± 2.1 h (90.6 HF)	8.7 ± 4.1 h (90.2 HF)	36.5 ± 4.9 f (61.7 MH)	41.8 ± 9.4 h (56.1 MH)
Neem	40.3 ± 7.3 c (55.0 MH)	41.8 ± 10.3 c (53.1MH)	74.1 ± 9.2 b (22.4 HL)	82.6 ± 16.2 c (13.3 HL)
Bittergourd	47.4 ± 9.7 b (47.1 SH)	49.2 ± 11.3 b (44.8 SH)	80.0 ± 9.8 b (16.2 HL)	88.7 ± 18.1 b (7.0 HL)
Control	89.5 ± 12.8 a	89.1 ± 16.2 a	95.5 ± 10.1 a	95.3 ± 19.2 a
P value	0.00003	0.0006	0.00002	0.0007
F value	3605.3**	3421.4**	1479.2**	2936.8**
*df*	6^a^/12^b^	6^a^/12^b^	6^a^/12^b^	6^a^/12^b^

Means with the same lowercase letters are not significantly different at α = 0.05 (Tukey’s test). Values in parenthesis indicate the corrected percent mortality of *E. formosa* inside the parasitized whitefly pseudopupae over control at different PAEI. HL, SH, MH and HF abbreviations inside the parenthesis indicate Harmless/Class-I, Slightly-harmful/Class-II, Moderately-harmful/Class-III and Harmful/Class-IV insecticides, respectively.

*df* Degree of freedom.

P, F and Df values = the respective times listed above.

^a^Degree of freedom of treatment.

^b^Error degree of freedom.

**Highly significant at probability level of 5%.

**Table 4:** Percent parasitism and adult emergence (mean ± SE) of *E. formosa* from parasitized pseudopupae and mortality (Mean ± SE) of adult and nymphs of whitefly and males and females of parasitoid (*E. formosa*) under laboratory conditions.

Treatments	Mortality of adult whitefly (%)	Mortality of whitefly nymphs (%)	Mortality of male *E. formosa* (%)	Mortality of female *E. formosa* (%)	Parasitism of whitefly pseudopupae by *E. formosa* (%)	Emergence of adult *E. formosa* from parasitized pseupupae (%) (% corrected mortality over control)
Pyriproxyfen	90.4 ± 7.53 a	88.8 ± 10.21 a	41.1 ± 4.13 e (SH)	35.6 ± 6.93 d (SH)	59.3 ± 7.45 ab	51.7 ± 10.52 cd (45.64% SH)
Buprofezin	90.5 ± 6.47 a	84.4 ± 9.91 a	46.6 ± 3.87 d (SH)	40.4 ± 7.75 c (SH)	54.7 ± 7.13 abc	47.6 ± 9.75 de (49.45% SH)
Fenoxycarb	85.7 ± 5.44 b	80.2 ± 9.01 a	56.5 ± 5.12 c (SH)	50.8 ± 8.03 b (SH)	44.4 ± 6.89 bc	41.9 ± 8.78 de (55.95% SH)
Methoxyfenozide	72.6 ± 6.13 c	66.5 ± 8.60 b	61.2 ± 5.51 b (SH)	55.4 ± 8.22 b (SH)	37.5 ± 5.12 cd	36.5 ± 9.02 de (61.62% SH)
Tebufenozide	65.1 ± 4.78 d	59.9 ± 9.21 b	76.3 ± 6.94 a (SH)	70.7 ± 9.51 a (SH)	25.6 ± 6.03 d	29.4 ± 8.53 e (69.09% SH)
Neem	45.2 ± 4.91 e	39.3 ± 6.78 c	36.3 ± 4.66 f (SH)	30.6 ± 5.72 d (SH)	64.2 ± 7.56 a	67.5 ± 10.14 bc (29.02% HL)
Bittergourd	35.3 ± 3.49 f	29.8 ± 7.13 c	26.5 ± 3.81 g (HL)	20.4 ± 5.01 e (HL)	67.5 ± 8.03 a	73.2 ± 14.81 b (23.03% HL)
Control	0.0 ± 0.00 d	0.0 ± 0.0 d	2.5 ± 0.7 h	0.0 ± 0.0 f	69.4 ± 9.34 a	95.1 ± 12.22 a
P value	0.00001	0.00004	0.0001	0.00002	0.001	0.0003
F value	1237.00**	1128.24**	4169.10**	453.44**	9434.52**	201.71**
*df*	7^a^/13^b^	7^a^/13^b^	7^a^/13^b^	7^a^/13^b^	7^a^/13^b^	7^a^/13^b^

Means with the same lowercase letters are not significantly different at α = 0.05 (Tukey’s test). Values in parenthesis indicate the corrected percent mortality of *E. formosa* inside the parasitized whitefly pseudopupae over control at 72 h PAEI. HL, SH, MH and HF abbreviations inside the parenthesis indicate Harmless/Class-I, Slightly-harmful/Class-II, Moderately-harmful/Class-III and Harmful/Class-I insecticides, respectively.

^a^Degree of freedom of treatment.

^b^Degree of freedom of error.

**Highly significant at probability level of 5%.

The results explained an exposure-interval dependent reduction in the whitefly population. The percent population reduction increased with an increase in PAEI of each biorational insecticide up to 72 h. Alternatively, a decreasing trend in percent whitefly population reduction was observed for PAEI of 168 h. Specifically, at all PAEI, pyriproxyfen demonstrated the highest percent whitefly population reduction and proved to be a more effective biorational insecticide than the other tested compounds, followed by buprofezin and fenoxycarb. Methoxyfenozide and tebufenozide were less effective than pyriproxyfen and buprofezin in reducing the whitefly population. Neem-extract and bitter gourd-extract were the least effective products, demonstrating a 26.6–41.6% and 21.5–36.5% reduction in whitefly populations, respectively; however, Neem-extract was more effective than bitter gourd-extract (Table 1).

Pyriproxyfen demonstrated approximately 12, 17, 23, and 20 times, buprofezin 11, 11, 20, and 19 times, fenoxycarb 10, 14, 18, and 15 times, methoxyfenozide 9, 13, 14 and 11 times, tebufenozide 7, 12, 13 and 10 times, Neem-extract 6, 11, 11 and 7 times and bitter gourd-extract 5, 10, 9 and 5 times more reduction in whitefly populations than the control at PAEI of 24, 48, 72 and 168 h, respectively (Table 1). A substantial increase in whitefly parasitism by *E. formosa* over time was recorded in all biorational insecticides treated plots.

Pyriproxyfen, buprofezin, fenoxycarb, methoxyfenozide, tebufenozide, Neem-extract, and bitter gourd-extract treated plots exhibited significantly lower whitefly parasitism at shorter PAEI (24 h) compared with higher whitefly parasitism at longer PAEI (168 h). The percent reduction in whitefly parasitism by *E. formosa* in all biorational insecticides treated plots was higher at 24 h PAEI; then, a considerable decline in parasitism reduction was recorded with increasing PAEI. Neem-extract, bitter gourd-extract, and pyriproxyfen proved moderately harmful to *E. formosa*, reducing parasitism to 51–75%, while buprofezin, fenoxycarb, methoxyfenozide, and tebufenozide were harmful and reduced parasitism by more than 75% at the PAEI of 24 h (Table 2).

At PAEI of 48 h, bitter gourd-extract was recorded as slightly harmful; Neem-extract, pyriproxyfen, and buprofezin were moderately harmful, whereas fenoxycarb, methoxyfenozide, and tebufenozide were attributed as harmful biorational insecticides. A comparison of IOBC toxicity scale with the calculated percent reduction in parasitism at PAEI of 72 h revealed that Neem-extract, bitter gourd-extract, pyriproxyfen, buprofezin, and fenoxycarb demonstrated less than 25% reduction in parasitism and were recognized as harmless/safe for *E. formosa*; while methoxyfenozide and tebufenozide attributing parasitism reduction between the range of 50–75% and > 75% were assorted as moderately-harmful and harmful insecticides, respectively. An increase in whitefly parasitism by *E. formosa* was observed in all plots treated with biorational insecticides (− 6.6 to − 15.4%) except in those plots which were treated with methoxyfenozide (43.3% parasitism reduction) and tebufenozide (63.3% parasitism reduction) at PAEI of 168 h. At this PAEI, pyriproxyfen, buprofezin, fenoxycarb, Neem-extract, and bitter gourd-extract were classified as harmless insecticides, while methoxyfenozide and tebufenozide were assorted as slightly harmful and moderately harmful insecticides, respectively (Table 2).

Percent emergence of adult *E. formosa* from field-collected parasitized whitefly pseudopupae varied significantly for different treatments at various PAEI (p < 0.05) (Table 3). An increasing trend in percent emergence and a decreasing trend in mortality of adult *E. formosa* was observed from those parasitized whitefly pseudopupae, which were collected at 24, 48, 72, and 168 h PAEI from cotton plots treated with all biorational insecticides. Neem-extract, pyriproxyfen, and buprofezin were found to be moderately harmful at 24 and 48 h PAEI as these demonstrated 22.5–41.8% emergence and a 53.1–74.9% mortality of adult parasitoids. However, fenoxycarb, methoxyfenozide, and tebufenozide had an 8.4–19.9% emergence and a 77.7–90.6% mortality of adult parasitoids (> 75%), showing them to be harmful. In comparison, bitter gourd-extract had an 47.4–49.2% emergence and a 44.8–47.1% mortality of adult parasitoid (between a range of 25–50%) and was shown to be a slightly harmful biorational at 24 and 48 h PAEI (Table 3).

At 72 and 168 h PAEI, pyroproxyfen, buprofezin, and fenoxycarb had an 48.3–69.5% emergence and a 27.1–49.4% mortality of adult parasitoids, showing them to be slightly harmful biorationals. Methoxyfenozide was slightly harmful (44.0% mortality) and moderately harmful at 168 h and 72 h PAEI, respectively. Tebufenozide had an 36.5–41.8% emergence and a 56.1–61.7% mortality of adult parasitoids, making it moderately harmful, while bitter gourd-extract and neem-extract contributed 74.1–88.7% emergence and a 7.0–22.4% adult parasitoid mortality, showing both to be harmless biorationals at 72 and 168 h PAEI (Table 3).

### Impact of biorationals under laboratory conditions

The treatments had a highly significant effect on variation in mortality of adults and nymphs of whitefly, mortality of parasitoid (*E. formosa*) males and females, and percent adult emergence of *E. formosa* from parasitized pseudopupae (p < 0.05) (Table 4). Pyriproxyfen (90.4% adults and 88.8% nymphs), buprofezin (90.5% adults and 84.4% nymphs) and fenoxycarb (85.7% adults and 80.2% nymphs) had mortality higher than 80% in whitefly adults and nymphs. In comparison, methoxyfenozide and tebufenozide had a 72.6 and 65.1% mortality in adults and 66.5 and 59.9% mortality in nymphs. Plant extracts had less than 50% mortality rates in whitefly adults and nymphs (Table 4).

The male and female parasitoids (*E. formosa*) released 24 h after the treatment application exhibited less than 50% mortality when exposed to pyriproxyfen (35.6% in females and 41.1% in males), buprofezin (40.4% in females and 46.6% in males), neem (30.6% in females and 36.3% in males) and bitter gourd (20.4% in females and 26.5% in males). Fenoxycarb, methoxyfenozide and tebufenozide demonstrated mortality in male and female *E. formosa* in the range of 50.8–76.3% (Table 4).

According to the mortality scale specified by IOBC to describe the effects of pesticides on beneficial species for laboratory, bitter gourd-extract proved harmless. In contrast, pyriproxyfen, buprofezin, fenoxycarb, methoxyfenozide, tebufenozide, and neem-extract proved slightly harmful for males and females individuals of *E. formosa*. Under laboratory conditions, more than 50% parasitism of whitefly pseudopupae by *E. formosa* (54.7–67.5%) was observed when pyriproxyfen, buprofezin, bitter gourd-extract, and neem-extract were applied 24 h before the release of the parasitoid. Statistically, similar parasitism was also recorded in the control treatment (69.4%). Fenoxycarb and methoxyfenozide demonstrated 44.4 and 37.5% parasitism, respectively, while tebufenozide resulted in the lowest amount of parasitism (25.6%) when applied 24 h before the release of the parasitoid. The data regarding the emergence of adult *E. formosa* from parasitized pseudopupae treated with insecticides indicate that bitter gourd and neem extract proved harmless (mortality < 30%) while pyriproxyfen, buprofezin, fenoxycarb, methoxyfenozide, and tebufenozide were slightly harmful (morality 30–79%) insecticides at parasitized pseudopupal stages (Table 4).

## Discussion

Only a few studies have focused on the importance of buprofezin, fenoxycarb, pyriproxyfen, methoxyfenozide, and tebufenozide (IGRs), Neem-extract and bitter gourd-extract in integrated management of *B. tabaci* in *Bt*-cotton. This is the first detailed study comparing the efficacy of these biorational insecticides against *B. tabaci* and evaluating their selectivity for its parasitoid, *E. formosa* on *Bt*-cotton. Whitefly, *B. tabaci* and its two potential parasitoid *E. formosa* and *Eretmocerus* species^1–4^ are targeted directly and/or indirectly through foliar application of conventional insecticides. These pesticides are intended to suppress *B. tabaci* but inadvertently reduce these key natural enemies' populations in the agro-ecosystem^63^. The laboratory and field studies in the present study demonstrate that adults and nymphs of *B. tabaci* show increased mortality in the laboratory and higher population reduction in field when exposed to pyriproxyfen followed by buprofezin, fenoxycarb, methoxyfenozide, tebufenozide neem-extract, and bitter gourd-extract. Similar findings were reported where buprofezin effectively controlled and significantly reduced the whitefly population in the field^4,63^. The present research results regarding the performance of pyriproxyfen and buprofezin against whiteflies were also consistent with the results that demonstrate the lower *B. tabaci* population in plots treated with buprofezin, pyriproxyfin, buprofezin + fenpropethrin, and pyriproxyfin + fenpropethrin (non-*Bt* cotton studies)^38,64^. However, the toxicity of tebufenozide, methoxyfenozide, fenoxycarb, and bitter gourd-oil cannot be compared or contradicted, as very little information is available in the literature reviewed.

Under field conditions, the reduction in the whitefly population increased with an increase in PAEI up to 72 h PAEI; however, a non-significant decreasing trend in population reduction in all treatments was observed in the field study at PAEI of 168 h. These results indicate that the efficacy of the tested biorationals started to decrease while the whitefly population flare-up, which may be attributed either to a reduction in the natural enemies’ population due to biorational stress or the emergence of a new population from pseudopupae in the treated plots. The results of commercial-scale field studies confirm that IGRs have very slow lethal impacts during the initial few hours of exposure. These lethal effects gradually increase and become significant within 1–3 weeks of post-application intervals because IGRs cause mortality in immature stages of *B. tabaci* only during ecdysis not before it^4,65,66^. The lethal effects of IGRs only on ecdysis of *B. tabaci* immatures are the possible reason behind the higher reduction in pest population during the longer post-exposure interval. This study also confirms that the tested IGRs proved effective against *B. tabaci*, causing a significantly higher reduction in its population (36.92–73.21%) up to 168 days (1 week). The observation on population reduction of *B. tabaci* in the field continued only for 168 h; hence, significant efficacy of IGRs reported for many weeks (2–5 weeks) by researchers^65,66^ cannot be discussed. The reduction in the whitefly population in the field up to 72 h PAEI may be attributed to a decrease in fecundity and egg-hatching as well as the mortality of nymphal stages during ecdysis due to IGRs^4,65,66^. The reduction in the efficacy of tested IGRs against *B. tabaci* at 168 days (1 week) in the present field study is attributed to prolonged exposure (168 days) of residues of applied IGRs to adverse field-experimental conditions (> 40 °C, < 55% RH), which result in steady photo-degradation (photolysis) of IGRs residues on the crop. Several factors like plant-species, plant development-stages, physiochemical properties of chemicals, formulation of chemicals, application methods, and climatic conditions (humidity, temperature, rainfall, wind-speed etc.) are responsible for the degradation and dissipation of agrochemicals (insecticides, fertilizers etc.) after their spray on plants^4,57^. It has been documented that a steady increase in temperature (beyond 30 °C) and a decrease in relative humidity (below 70%) result in the gradual degradation of residues of IGRs, reduction in their half-life, and resultantly reduction in their efficacy^4,57,58^. A variation in the toxicity/performance of tested biorationals (five IGRs and two plant-extracts) in present laboratory and field studies may be attributed to differences in experimental conditions experienced by the target insect (*B. tabaci*) and biorational molecules in the laboratory (30 ± 2 °C, 65 ± 5% RH) and field (> 40 °C and relative humidity fluctuating between 50 and 70% in cotton crop). Higher mortality in laboratory study may be due to the fact that the *B. tabaci* population under laboratory conditions was exposed to treated plants where they were forced to perform their life activities under no-choice bioassays, unlike the field study, where the population was exposed to abiotic conditions (temperature, humidity, and rainfall), exhibiting lower population reduction.

The results regarding the effects of tested biorationals to *B. tabaci* parasitoid (*E*. *formosa*) indicate that percent parasitism increased, percent mortality of *E. formosa* inside host-pseudopupae decreased, the percent reduction in parasitism declined, and the toxicity class of tested insecticides changed from harmful to harmless with an increase in PAEI from 24 to 168 h. An increase in parasitism and a decline in the reduction of parasitism of *B. tabaci* pseudopupae by adult *E. formosa* exhibited in the present study may be due to insecticides losing their contact toxicity with an increasing exposure interval of 168 h. This is confirmed by the declining trend in population-reduction and population flare-up of whiteflies at this exposure interval (168 h) in the present study. This steady reduction in contact toxicity of tested chemicals and flaring up of host (whitefly) population (as observed in the present study) enhance the survival rate of parasitoids, encourage the availability of sufficient host and ultimately result in a higher parasitism rate. The tested IGRs are recommended against insect pests on crops where these IGRs absorb into and form a reservoir of their toxic residues within the leaf-tissues (local-systemic/translaminar action). This type of absorption of IGRs residues increases with post-application interval and hence minimizes their residual toxicity on the treated leaf-surface^4,57^. Such types of post-application behavior of IGRs molecules ensure a gradual increase in toxicity against sucking pests like whiteflies (but not longer than 1–2 weeks) and a steady decrease in contact toxicity against predators, parasitoids^57^ and other non-target arthropod fauna^4^. The selectivity of the tested biorational insecticides to the parasitoid, *E. formosa,* may be related to the difference in their dose rate, formulations and mode of action, the parasitoid's exposed stage, and post-application exposure interval^4,64,67^.

At the maximum PAEI in this study (168 h), methoxyfenozide was slightly harmful, and Class-II insecticide (parasitism reduction between 25 and 50%) and tebufenozide was moderately harmful and Class-III insecticide (parasitism reduction between 50 and 75%). Alternatively, three IGRs (buprofezin, fenoxycarb and pyriproxyfen,) and two oil-extracts of neem and bitter gourd seeds, were harmless and Class-I insecticides as these biorationals demonstrated less than 25% reduction in parasitism in the field study. This reduction in parasitism would not be considered significant based on IOBC standards specified for field studies^59^. There is little information available in the literature regarding the categorization of insecticides based on parasitism reduction percentage of *E. formosa*. However, in another study, it was reported that methoxyfenozide (24 EC @ 36 g a.i./200 L H_2_O) did not affect adults of the egg parasitoid *Telenomus remus* (Nixon) when tested through glass-plate bioassay under laboratory conditions (25 ± 1 °C, 70 ± 10% RH, with a 14/10 h (L/D) photoperiod)^68^. Similarly, the application of methoxyfenozide at its field recommended dose (39.8 g a.i./100 L H_2_O) proved non-toxic to *Trichogramma* nr. *brassicae* (egg parasitoid of *Helicoverpa* species) in both laboratory (filter paper bioassay conducted at 25 °C in plastic Petri dish under Potter Spray Tower against female parasitoid chilled at 4 °C for 3 min) and field trials (foliar spray with by Silvan battery powered backpack sprayer with T-jet cone nozzle on tomato beds), as it demonstrated no harmful effects on adult emergence from sprayed parasitized eggs of *T.* nr. *brassicae*^69^. These results are not in agreement with our findings about methoxyfenozide, which was categorized as a slightly-harmful biorational insecticide against *E. formosa* because methoxyfenozide caused mortality of parasitoids between a range of 25–50% in field-study and 30–79% in lab studies. These mortality ranges fall in mortality scale specified by the IOBC for slightly-harmful/Class-II insecticides against beneficial species for field and lab studies^59^. These variations in results may exist due to difference in the concentration/dose-rates of methoxyfenozide (200 mL/0.4 ha), materials and methods used in the field (foliar spraying by 3.5-gallon knapsack sprayer with a Teejet hollow cone spray cores D3 disk using 1380-L of water/ha) and lab (foliar application by an atomizer on potted cotton maintained at 25 ± 2 °C and 75 ± 5% RH) studies, choice of test parasitoid (*E. formosa*), and the life stage (adult of *E. formosa* and unparasitized pseudopupae and larval instars of *B. tabaci*) used in our experiments.

Some studies demonstrate that different development stages of Hymenopterous parasitoids respond differently when exposed to IGRs’ treatments^47^. There is a variable response to different stages of parasitoids to insecticides and IGRs. The effects of these compounds on predators/parasitoids are contradictory and inconsistent^46,47^. The results of the present study show that at the maximum PAEI (168 h), pyriproxyfen, buprofezin, fenoxycarb, and methoxyfenozide were in Class-II biorationals (slightly harmful, 25–50% mortality), neem and bitter gourd oils were Class-I biorationals (harmless, < 25% mortality), and tebufenozide was in Class-III biorationals (moderately harmful, 50–75% mortality) when emergence and mortality of *E. formosa* were recorded inside the treated host pseudopupae in lab bioassay. In parasitized pseudopupae-exposure bioassay (field), neem and bitter gourd oil were demonstrated as harmless/Class-I biorationals (< 30% mortality), while pyriproxyfen, buprofezin, fenoxycarb, methoxyfenozide, and tebufenozide were confirmed as slightly-harmful biorationals (30–79% mortality) against *E. formosa* stages developing inside host pseudopupae. Our results regarding pyriproxyfen are not in agreement with other findings, which confirm pyriproxyfen as highly harmful to larvae but moderately harmful to pupae of *E. formosa*^46,48^*.* Similarly, present results regarding buprofezin are contradictory to the results which demonstrate buprofezin as harmless to *E. formosa* pupae^46^*.* However, our findings regarding fenoxycarb corroborate other studies indicating that fenoxycarb is slightly harmful IGR to larvae and pupae of *E. formosa*^46^ and *Eretmocerus eremicus*^70^*.* Our findings regarding the toxicity of methoxyfenozide and tebufenozide to *E. formosa* stages developing inside host pseudopupae cannot be compared or contradicted as no information is available in reviewed literature in this line of study. The adult-exposure lab bioassay demonstrated that all tested biorationals except bitter gourd oil (harmless/Class-I) were classed as slightly-harmful biorationals (30–79% mortality) against male and female *E. formosa* adults; however, female-adults exhibited lower mortality than the male-adults. Our lab bioassay results regarding pyriproxyfen, buprofezin and fenoxycarb are highly in accordance with the results that demonstrate these three IGRs as slightly harmful to *E. formosa* adults in glass-vial bioassay^46^. The reasons behind the higher mortality of *E. formosa* male-adults than its female-adults has not been investigated in the present study. Azadirachtin-A, azadirachtin-B, nimbina and salamina at 9.6 ppm were in general safe (Class-1) to both adults and pupae of *Trichogramma pretiosum*^71^. These botanicals did not affect *T. pretiosum* and proved highly compatible with this parasitoid. NEEMSETO proved safe for *T. pretiosum* in the first 24 h^72^. These results are highly consistent with our findings regarding neem oil extract (harmless). Azadirachtin, buprofezin, tebufenozide, and pyriproxyfen are harmless to parasitoids and slightly detrimental to parasitoid emergence^64,67,73^. The present field experiment results indicate that pyriproxyfen, buprofezin and fenoxycarb are harmless to parasitoids and slightly detrimental to the emergence of adult *E. formosa* from parasitized pseudopupae at 72 h PAEI. These results demonstrate that these IGRs, especially pyriproxyfen, can be used in integration with *E. formosa*. Our field bioassay findings regarding pyriproxyfen, buprofezin and fenoxycarb are highly in accordance with the results that demonstrate these three IGRs as harmless to *E. formosa* adults on tomato leaves in field bioassay^46^. Similarly, different researchers' findings on buprofezin and *E. formosa* compatibility confirm that leaf residues of buprofezin are not detrimental to *B. tabaci* parasitoids (*Encarsia* and *Eretmocerus* species) as it does not affect the foraging of adults parasitoids of these species on treated leaves^74–77^. These findings^74–77^ also confirm our results concerning buprofezin as harmless IGR to *E. formosa* adults in field bioassay.

Augmentation of natural enemies (predators and parasitoids) for biological control of pests may play an imperative role in integrated pest management programs/systems. But chemical control (insecticides), a vital component of pest management, disrupts bioagents’ performance in the biological control system of pests^4,46–48,58,78^. However, IGRs are significant components of chemical control that suppress the pests effectively and are relatively safer for their natural enemies^4,46–48,58,70,78^. A compatibility ranking of buprofezin > fenoxycarb > pyriproxyfen > kinoprene was reported against *Er. Eremicus* (a parasitoid of whitefly)^70^. However, the compatibilities of tested biorationals to whitefly parasitoid *E. formosa* in present study were ranked as bitter gourd-extract > neem-extract > pyriproxyfen > buprofezin > fenoxycarb > methoxyfenozide > tebufenozide; while toxicity to whitefly *B. tabaci* was ranked as pyriproxyfen > buprofezin > fenoxycarb > methoxyfenozide > tebufenozide > neem-extract > bitter gourd-extract.

*Bt*-cotton has a different production technology regarding pesticide, fertilizer (nutrient), and water requirements. These factors influence various biotic stresses and exogenic molecules differently due to differences in their genetic background and physiology/metabolism^22^. Some of the differences in the efficacy of the insecticides tested may be attributed to differences in these ecological requirements for *Bt*-crops. These variations in the agronomic technologies may affect the quantity and quality of specialized-metabolite biosynthesis differently in *Bt* and non-*Bt* crops^23,24^, which may affect a pesticide's performance on a sucking pest such as *B. tabaci*^25^.

The present study results affirm that most of the tested biorationals (compounds) are moderately harmful to harmful within the first 48 h PAEI. Afterward, these molecules are classed as slightly harmful to harmless biorationals. These results highlight the most appropriate PAEI of these compounds before and after which these molecules are compatible with *E. formosa.* It is evident from these results that effective integration of pyriproxyfen, buprofezin, and fenoxycarb with *E. formosa* can be achieved if either these IGRs are sprayed at least 72 h before augmentation of *E. formosa* or *E. formosa* is augmented after 72 h of sprays of these IGRs. However, there is a need to investigate and reconfirm it in an IGR and *E. formosa* based IPM experiment with different PAEI ranging from 3 to 15 days. In the present study, compatibility effects of these biorationals were assessed only on one whitefly parasitoid i.e., *E. formosa*, which is one component of bioagents-complex of *B. tabaci* in *Bt* cotton. So, it is vitally imperative and essential to validate the compatibility effects of these biorationals against other predator and parasitoid species of whitefly under lab, semi-field and field conditions to come up with a sound and effective biorational-bioagent based IPM program for whitefly.

## Conclusions

The present study results affirm that most of the tested biorationals (compounds) are relatively more toxic within the first 48 h PAEI and afterward prove comparatively safer to *E. formosa*. These results also define an effective PAEI (at least 72 h) for comparatively safer biorational molecules. Most of the tested biorationals proved harmless or slightly harmful to *E. formosa*, except tebufenozide after PAEI of 7 days in the field. Hence, they can be exploited strategically in *B. tabaci* management program. Overall, the compatibility ranking of tested biorationals to *E. formosa* was bitter gourd-extract > neem-extract > pyriproxyfen > buprofezin > fenoxycarb > methoxyfenozide > tebufenozide; while their order of toxicity to *B. tabaci* was pyriproxyfen > buprofezin > fenoxycarb > methoxyfenozide > tebufenozide > neem-extract > bitter gourd-extract. These two rankings indicate that pyriproxyfen, buprofezin, and fenoxycarb proved effective against *B. tabaci* and are relatively more compatible with *E. formosa.* Hence, effective integration of these three molecules with *E. formosa,* and an efficient *E. formosa* supplemented augmentative biocontrol system can be established either by devising a spray-schedule strategy of these three IGRs at least 72 h before augmentation of *E. formosa* or by planning *E. formosa* augmentation after 72 h of sprays of these IGRs. Nonetheless, it is emphasized that an IGR and *E. formosa*-based IPM experiments should be conducted at PAEI, ranging between 3 and 15 days to define IGR and *E. formosa*-based efficient strategy for effectual management of *B. tabaci*.

## Data Availability

The datasets used and/or analyzed during the current study are available from the corresponding author on reasonable request.
